# Amber imitation? Two unusual cases of *Pinus* resin-coated beads in Iberian Late Prehistory (3^rd^ and 2^nd^ millennia BC)

**DOI:** 10.1371/journal.pone.0215469

**Published:** 2019-05-03

**Authors:** Carlos P. Odriozola, José Ángel Garrido Cordero, Joan Daura, Montserrat Sanz, José María Martínez-Blanes, Miguel Ángel Avilés

**Affiliations:** 1 Departamento de Prehistoria y Arqueología, Universidad de Sevilla, C/ María de Padilla S/N, Sevilla, Spain; 2 Grup de Recerca del Quaternari (GRQ-SERP), Departament d'Història i Arqueologia, Universitat de Barcelona, Carrer Montalegre, 6, Barcelona, Spain; 3 Instituto de Ciencia de Materiales de Sevilla, Centro mixto CSIC-Universidad de Sevilla, Sevilla, Spain; University at Buffalo - The State University of New York, UNITED STATES

## Abstract

A group of beads from the artificial cave of La Molina (Lora de Estepa, Sevilla) and Cova del Gegant (Sitges, Barcelona) were made from a biogenic raw material and intentionally covered by a layer of resin. This is the first time this type of treatment has been documented on elements of adornment in the Late Prehistory of the Iberian Peninsula. The composition and nature of the coatings are analysed and the symbolic role of such alterations and imitations of prehistoric adornments is discussed.

## Introduction

Since the Upper Palaeolithic, the unique translucent reddish-yellow colour of amber has made this resinite sought after for personal ornamentation, e.g. beads, pendants, charms… [1], conferring on an important symbolic value in European Prehistory. The allure and rarity of amber triggered the exchange and use of this resinite, but also the development of imitations by the use of other local translucent minerals [2] or the application of coatings, as described by this paper, to reproduce the colour of amber.

The utmost importance to prehistoric societies of colour categories and their impact to show, emphasize and materialize codes, metaphors and narratives has been highlighted by several authors [3–10]. Although altering the genuine colour of raw materials or products by means of technically controlled processes to obtain a desired effect seems to have been a usual practice in past societies, this technical behaviour has been occasionally recorded and studied.

Colour modification practices in personal ornaments goes back to the Upper Palaeolithic, when in Franchthi Cave (Greece) perforated shell beads were thermally altered to obtain black hues [11]. A similar intentional effect has been also recorded in discoidal beads made from shells at the Chalcolithic site of Perdigões (Portugal) [12]. Colour modification by heat has also been recorded in carnelian quartz beads in the Varna necropolis (Bulgaria), creating darker hues that were complemented with a complex faceting technique to enhance surface gloss [13]. However, during the Neolithic much more complex processes of thermal colour transformation appeared. The application of different kinds of coatings became common to obtain specific performance characteristics and display desired new colours of rare raw materials in personal ornaments. This is the case at Tell el-Kerkh (Syria) where during the 6^th^ millennium BC the highly appreciated turquoise blue beads [14] were imitated by thermal alteration of apatite in presence of a transition metal to produce odontollite [15], which was further fixed to the beads with a natural resin. Similar examples shows the success of this technique and the display of imitation turquoise among contemporaneous Neolithic communities in the Near East [16]

**Fig 1:**
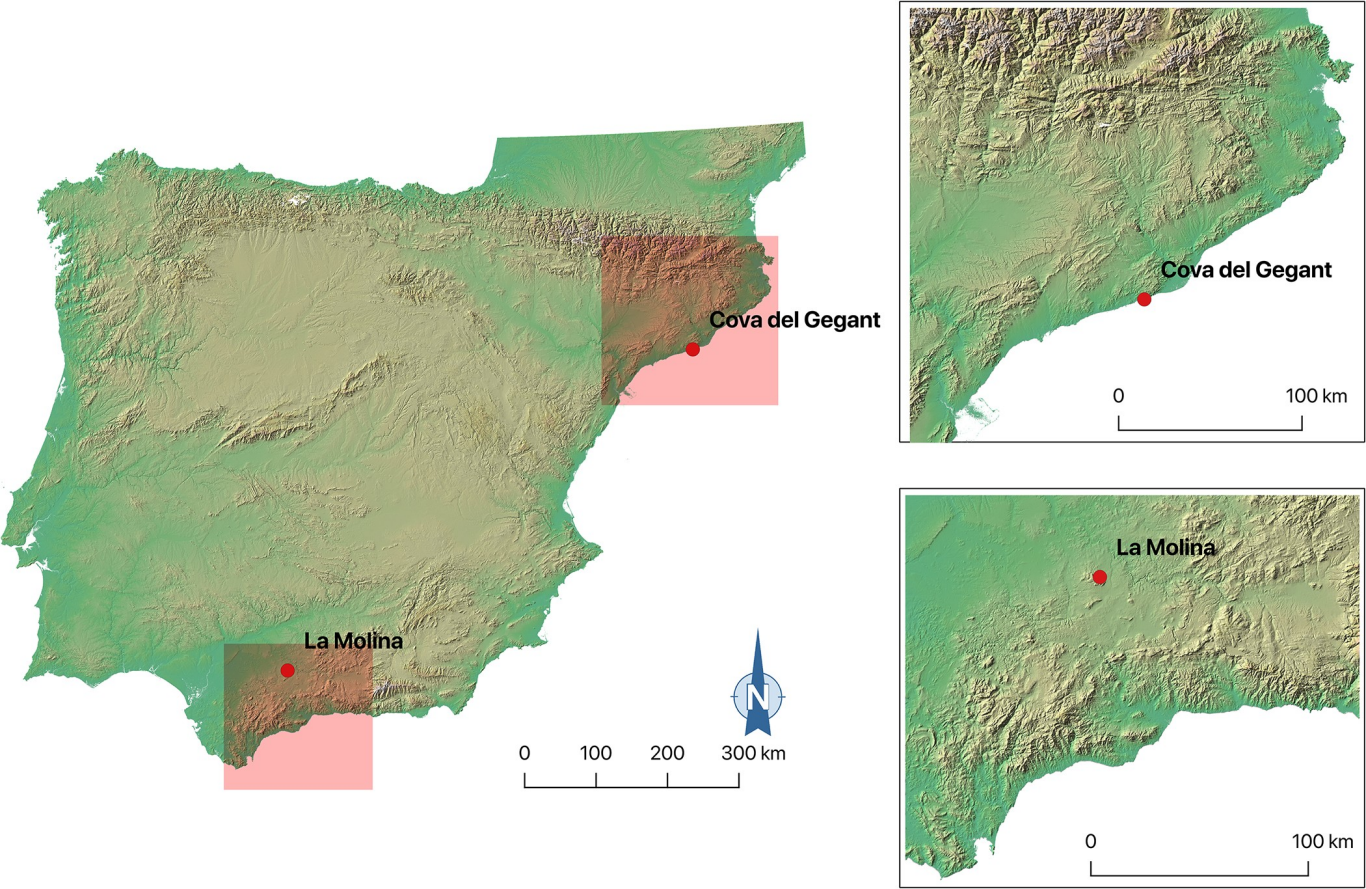
Location of the studied sites.

This paper analyses some unusual items from the funerary contexts of La Molina (Lora de Estepa, Sevilla, 3^rd^ millennium BC) and Cova del Gegant (Sitges, Barcelona, 2^nd^ millennium BC) (Fig 1). In all the case studies, biogenic beads have been coated with a resinite that resembles the appearance of amber beads. These amber resembling beads and their coating technique are currently the only cases known for European Late Prehistory.

First, the nature and technology of the bead cores and coatings are characterised by means of electron microprobe (SEM-EDX), infrared spectroscopy (FTIR), x-ray diffraction (XRD) and Raman spectroscopy. Then the role of raw materials as exotica is discussed, considering the importance of imitations in cases where procurement of highly appreciated raw material is disrupted by political or economic factors.

## Case studies

### La Molina (Lora de Estepa, Sevilla)

**Fig 2:**
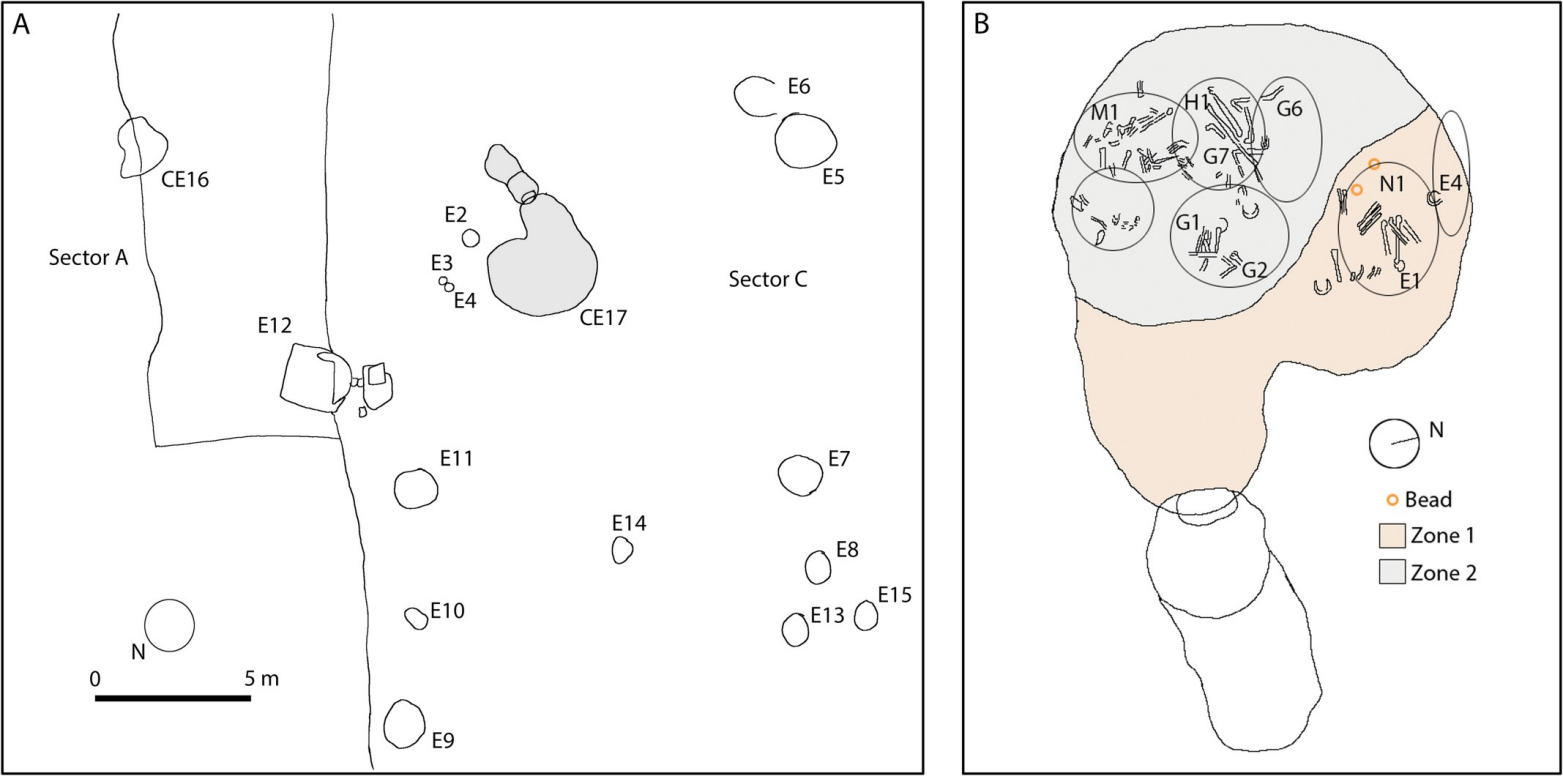
A. general plan of the excavated area at La Molina (After Fig 4.2 in [17]). B. Human remains of La Molina´s artificial cave CE17. Indicated by circle/ellipsis and labels are bone assemblages and individuals (After Figs 4.3 to 4.6 in [17]).

The artificial cave CE17 (Fig 2) at La Molina was discovered in 2008, next to another hypogean burial that was partially destroyed (CE16) and a series of negative structures in the form of silos [17]. The structure consists of a small descending corridor 1.5m long with two steps, leading to the entry of an oval-shaped chamber with a maximum diameter of 3.5m [18]. The mortuary space, which was divided into two areas that were differentiated both spatially and in the funerary treatment, contained a minimum number of ten individuals [18]. The grave goods consisted of several pottery vessels, bone awls and singular objects in ivory, as well as abundant chipped and polished lithic artefacts, particularly two arrowheads in rock crystal [19]. The excavators dated this mortuary site in the early third millennium BC by material and contextual comparisons after absolute dating failure [18].

Two beads with a more or less discoidal shape (Fig 5.23 in [19] were found in association with the mortuary layer of stones called Structure III (SU 106a) [18]. This part of the chamber (Zone 1) contained practically all the cinnabar in the tomb, where it was found on all the individuals (MNI = 3), compared with Zone 2, where it was only occasionally found. It also contained most of the ivory in the tomb [19], attesting the differential and special treatment of the individuals buried in this part of the chamber in comparison with those in Zone 2. The individuals (N1, an adult male in anatomical connection; E1, a young adult female; and E4, an adult male) were deposited in the Zone 1 near the chamber wall on top of some prominent stones [18]. Only E1 possessed grave goods unmistakably placed near her head, including a carved elephant’s tusk, a flint knife with ivory handle, and other ivory and lithic artefacts, which indicate the prestige of the inhumation.

### Cova del Gegant (Sitges, Barcelona)

**Fig 3:**
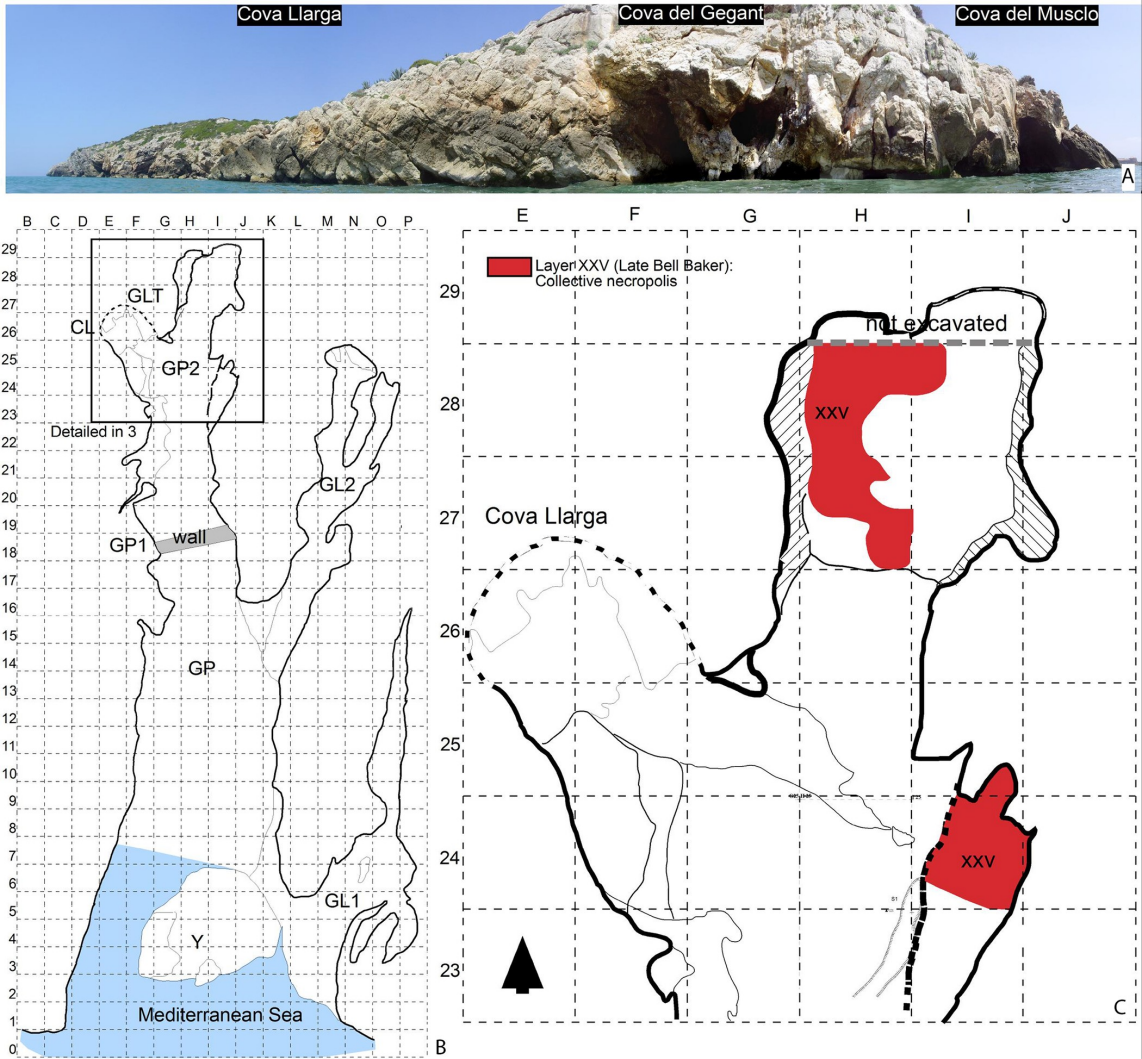
A. Site plan of the Cova del Gegant indicating the position of the different galleries mentioned in the text. B. Location of the excavated area. C. Stratigraphy (section CC’ as detailed in B) (After Fig 1 in [20].

Cova del Gegant (Fig 3) is a cave located in the NE part of the Iberian Peninsula, ~40 km south of Barcelona (1° 46’ 27.33”E, 41° 13’ 24.75”N, 0m abmsl). It consists of a main chamber (GP), now eroded by wave action, and its inner part (GP2), where a small passage (GLT) leads to the adjacent Cova Llarga. Two galleries branch off on the right side of GP, one towards the interior (GL2) and the other nearer to the sea (GL1). The original entrance is partially flooded and faces the sea.

At least eight site formation episodes from the Upper Pleistocene to the Holocene have been recorded in the Cova del Gegant stratigraphic sequence [21]. As regards the Holocene layer, Episode 4 is the oldest (Bronze Age), represented by Levels VI and Ic2 (Sectors GP and GL2) and Layer XXV (Sector GP2) with which the beads are associated (Fig 3, Table 1). Two additional pit-storages (Silo-1 and Silo 2) have been documented in the same formation episode [21].

**Table 1:** Brief description of the beads studied in this paper (H: height, W: width, P: perforation diameter, Wg.: weight).

# ID	Site	Core	H	W	P	Wg.	Color	Chronology	Arch. context
4316	Cova del Gegant	Shell	2.07	8.04	2.62	0.22	Reddish yellow	Early 2^nd^ millennium BC	Natural cave, col. burial
4472	Cova del Gegant	Shell	2.11	8.12	2.61	0.21	Reddish yellow	Early 2^nd^ millennium BC	Natural cave, col. burial
4473	Cova del Gegant	Shell	1.93	9.52	2.63	0.12	Reddish yellow	Early 2^nd^ millennium BC	Natural cave, col. burial
4476	Cova del Gegant	Shell	2.16	6.20	2.46	0.11	Reddish yellow	Early 2^nd^ millennium BC	Natural cave, col. burial
M-89	La Molina	¿?	5.55	7.82	2.41	0.16	Red	Early 3^rd^ millennium BC	Artificial cave, col. burial
M-90	La Molina	¿?	3.30	13.63	2.43	0.22	Red	Early 3^rd^ millennium BC	Artificial cave, col. burial

The archaeological layer ascribed to the Bronze Age (XXV), mainly preserved at the back of the main gallery (GP2), corresponds to the grid delimited area between squares H27 and H28 (~1.8 m^2^) [20]. To the south, archaeological layer XXV is not preserved due to the ongoing marine erosion. This layer corresponds to a collective burial accumulation radiocarbon dated to the Middle Bronze Age, 1600–1400 BC. Among the archaeological remains, the bulk of the assemblage is formed by 1,728 human bones ascribed to a Minimum Number of Individuals (here after MNI) of 19 disarticulated individuals. The artefacts accompanying the recovered human remains comprise Late Bell Beaker pottery (NR = 71) generally ascribed to the Early Bronze Age. Body ornaments are scarce and consist of beads manufactured from a variety of raw materials. For example, three of them are made of lignite, two of amber [2], one of coral and one of a shell fragment (*Cypraea)*. Four beads were manufactured from shell and covered by a resinite; these are the pieces analysed in the present study (Table 1). The Sicilian origin of the amber materials evidences that the area participated in long-distance exchange networks [2]. Additionally, the two gold artefacts (*tutuli*) recovered from the same layer are very rare ornaments in the SW of Europe and reinforce the idea that the site formed part of an exchange network along the Mediterranean shore of the Iberian Peninsula [20].

## Materials & methods

Administrative permits to study La Molina beads were granted to Carlos P. Odriozola and José Ángel Garrido Cordero by the Junta de Andalucía Delegación territorial de Educación Cultura y Deporte, Museo Arqueológico de Sevilla (DCI/ES/2014/19). The ID numbers in Table 1 are those used by the Museo Arqueológico de Sevilla (Sevilla, Spain) to label the material. Archaeological excavations and beads sampling from Cova del Gegant are approved by the project “Els canvis climàtics durant el plistocè superior a la costa central catalana i l'impacte en les poblacions neandertals i humans anatòmicament moderns” (CLT009/18/00022, Generalitat de Catalunya). The ID numbers for Cova del Gegant materials in Table 1 are those given by the site inventory number. These materials are housed at the University of Barcelona.

**Fig 4:**
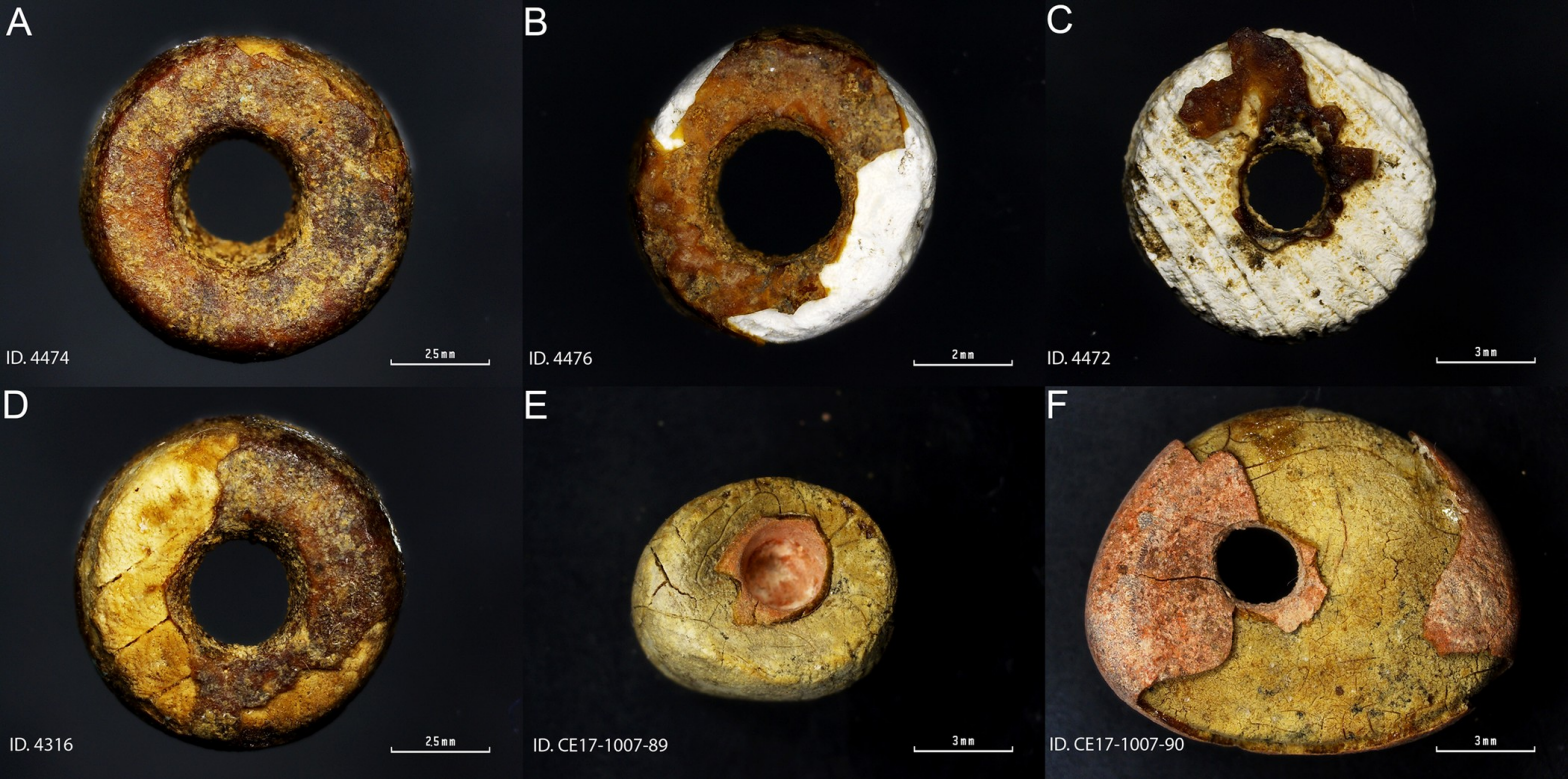
Samples studied in this paper.

Infrared spectroscopy (FTIR), Scanning Electron Microscope Microprobe (SEM-EMP), x-ray diffraction (XRD) and confocal dispersive μ-Raman spectroscopy (DcμRS) were used to investigate the nature and technology of the coated beads from both sites (Table 1, Fig 4). These instrumental techniques are able to obtain full details of the chemical composition and structure of both the bead core and the coating, with the double aim of understanding their nature and technology.

Infrared spectroscopy is capable of satisfactorily identifying resinites, their composition and botanical origin, to classify them and can be performed either non-destructively, by means infrared microscopy (μ-FTIR), or destructively on a very small sample of no more than 1 mg. It has become the standard technique applied in archaeological research to determine the nature and origin of resinites. We have therefore followed this well-known methodology [22] to study the six amber-like (resinite) coated beads.

Approximately 1mg of sample was ground by hand using an agate mortar and mixed with a small amount of KBr, before pressing (8 T) the mixture to produce discs 1mm thick. The specimens were analysed using a JASCO FT/IR-6200 spectrometer. The data were collected as infrared transmission spectra after scanning each specimen 32 times in the range 4000–400 cm^−1^, with a resolution of 4 cm^−1^.

X-ray diffraction is an inexpensive high-resolution technique which provides information about the mineralogical composition. It has been used to identify the pigments the coatings are composed of. After the baseline calculation with the X’Pert Highscore Plus 3.0 software, and when all the peaks in the diagram have been identified, the numerical values obtained were compared with the 2004 ICDD (International Centre for Diffraction Data) PDF (Powder Diffraction File) database, with the aim of identifying the minerals that the sample is composed of.

A Panalytical X’Pert Pro θ/θ X-ray diffraction equipment with Cu Kα (1.5406 Å) radiation operated at 45 kV and 40 mA, equipped with a PixCel detector and parabolic incident beam mirrors was used. The diagrams were acquired with a step of 0.026° 2θ between 10° and 90° 2θ with an acquisition time of 247 s per step at room temperature (25°C).

Confocal dispersive μ-Raman spectroscopy, also a non-destructive technique, is used to identify solids through vibrations in the crystalline lattice, as it can detect the sample composition, bonds, coordination environment and crystalline structure [23–24].

The data was obtained with a HORIBA Jobin Yvon LabRAM HR dispersive confocal μ-Raman spectrometer system. The laser diode, when operated at a wavelength of 532.06 nm, produces up to 15 mW of power in the source. Filters to reduce the laser’s power were not used. The acquisition time was 32 s per acquisition, up to a maximum of 20. The spectral measurement range chosen was between 100 and 1800 cm^-1^ using a 100x lens with a CCD multi-channel detector. The selected measurement is accurate to 1 cm^-1^. The measurement area selected was 1000 μm in diameter.

A Hitachi S4800 high resolution (1–3 nm) field emission scanning electron microscope, equipped with a 1.33 eV resolution Bruker X Flash 4010 EDX detector equipment was used for the composition analysis.

Additionally, routine close-up inspection of the bead coatings with a Nikon Shuttlepix P-4000R digital microscope (up to x80 magnification) was performed to gain full image details of the bead coatings.

This analytical study was conducted jointly at the Institute for Material Science in Seville (CSIC–University of Seville) and the Prehistory and Archaeology Department (University of Seville) laboratories.

## Results

### Cova del Gegant

**Fig 5:**
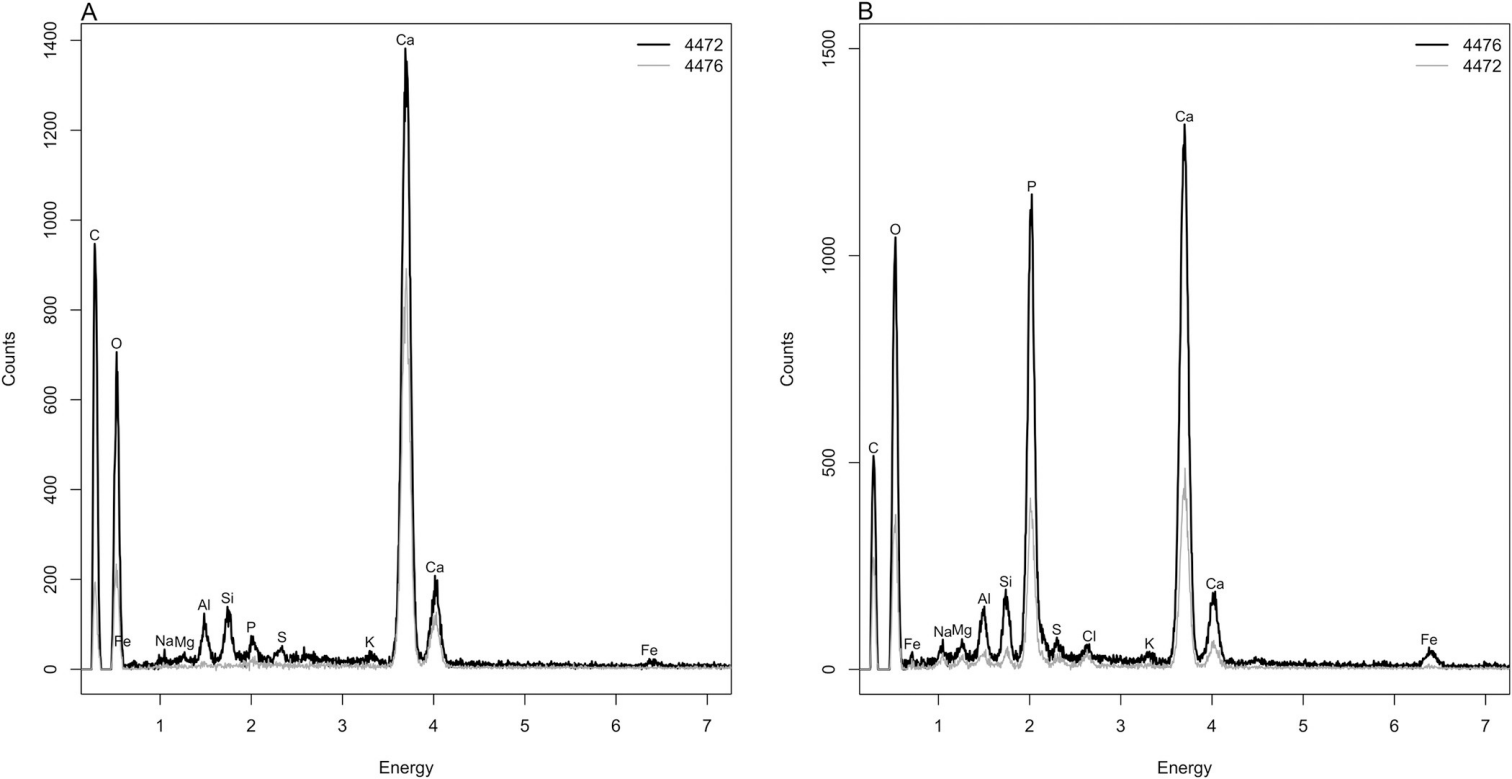
Cova del Gegant beads 4472 and 4476 SEM-EMP analysis of the bead core (A) and superficial coating (B).

Taking advantage of the cracked coating that leaves the beads’ white core exposed, SEM-EMP analysis was performed on both the surface of the white core and the surface of the reddish-yellow coating. The elemental analysis revealed that the core of the beads is mainly composed of calcium (Fig 5A). While when performed on the bead coating surface, it revealed a mixture of calcium and phosphorus (Fig 5B).

**Fig 6:**
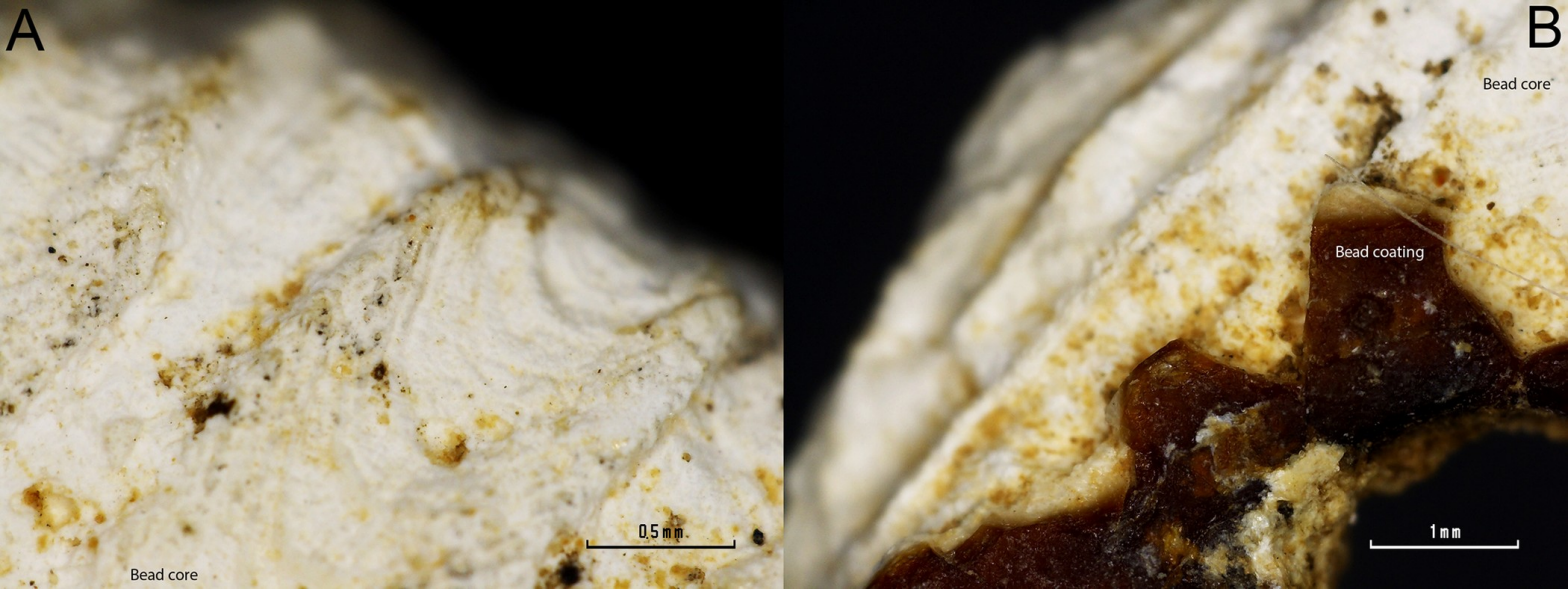
Detailed microphotographs of bead 4474 in a region where the coating is lost. A) Detail of the radial ribs of a mollusc valve. B) Detail of the conserved coating.

Both, the radial ribs of a mollusc valve (4474) shown in Fig 6, and the chemical composition of the bead core, points to the use of a valve for the bead making. Most mollusc shells are made of aragonite, a type of calcium carbonate [CaCO_3_].

**Fig 7:**
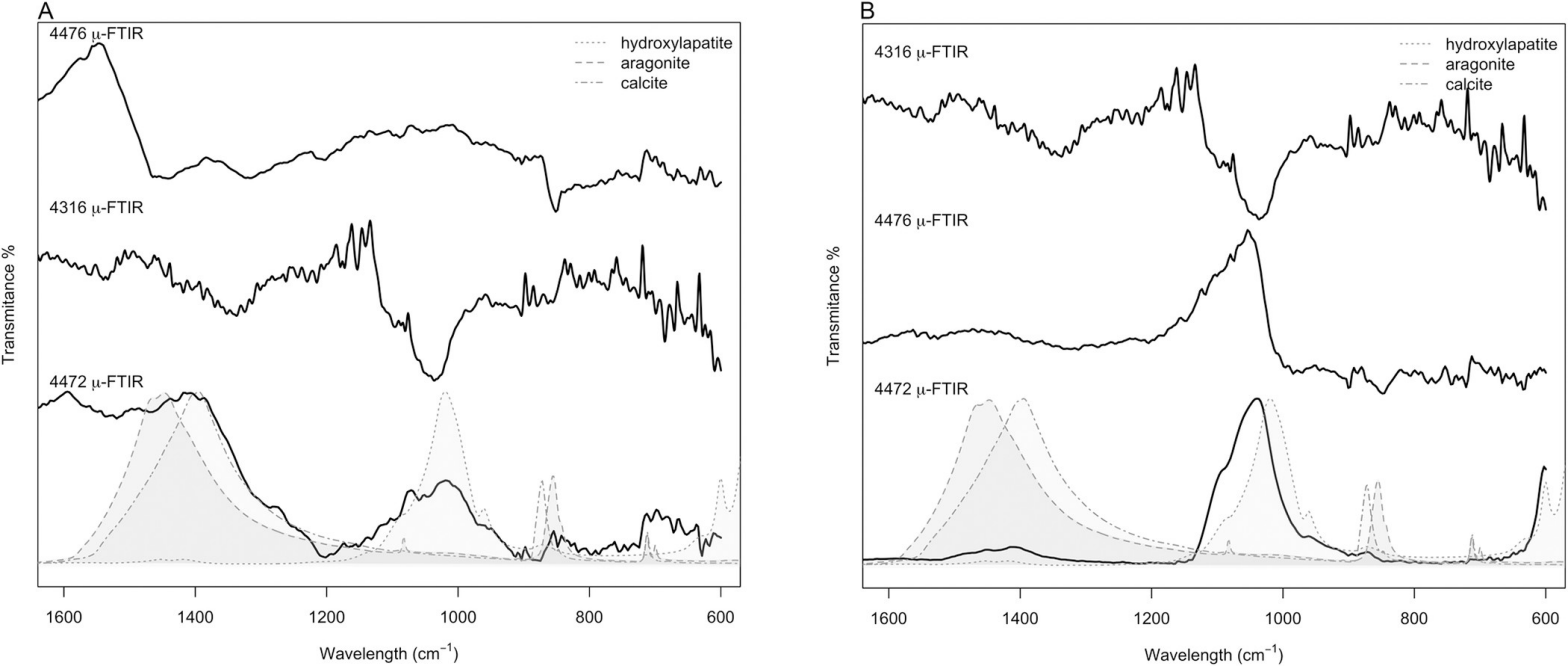
Micro FTIR spectra of (A) white core surface. (B) coating surface.

FTIR microscopy of the surface of the white core material showed, for specimens 4472 and 4316, the presence of a band centred at *c*. 1087 cm^-1^ and a band at *c*. 1488 cm^-1^ assigned to the υ_1_ and υ_4_ symmetric stretching modes of the aragonite [25] (Fig 7A). The band at *c*. 1087 cm^-1^ is active only in aragonite and is not active in calcite; it has therefore been used to identify the nature of the white calcium carbonate core. The presence of these aragonite assigned bands suggests that a mollusc was used to produce the core of the coated beads from Cova del Gegant.

FTIR microscopy of the surface of the reddish-yellow coating (Fig 7B) yielded similar spectra to those for the white core of the beads. μ-FTIR identified υ_3_ and υ_1_ hydroxyapatite vibration modes [26], together with υ_1_ symmetric stretching mode of the aragonite [25], and calcite’s υ_3_, υ_2_ and υ_1_ vibration modes [25] overlapped by other signals that remain unassigned.

**Fig 8:**
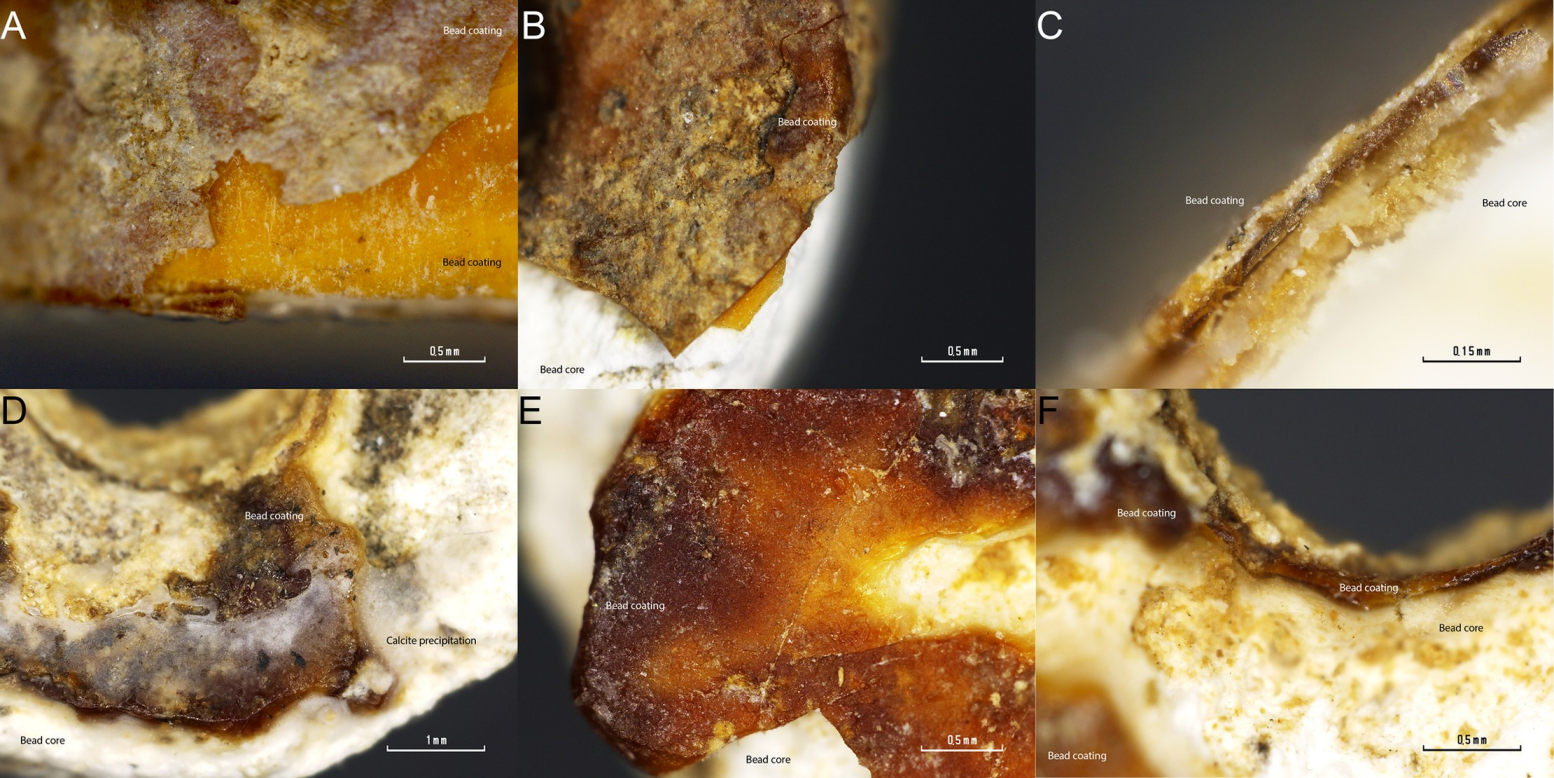
(A-C) detailed imaged of the 4472 bead coating. A white layer can be observed over a reddish-yellow one. (A) Zenithal view of the bead surface. (B) Detailed view of the surface coating. (C) Section view of the coating. (D-F) Detailed imaged of the 4474 bead coating. A white layer can be observed over a reddish-yellow one. (D) Zenithal view of the bead surface. (E) Detailed view of the reddish amber-like layer. (F) Section view of the coating.

FTIR band assignment suggests that the white outer layer is most likely the result of taphonomic processes. The basic pH of the cave sediment, *c*. 8.4 [2], has dissolved the hard bone tissue of the buried humans which have subsequently precipitated as some sort of apatitic calcium phosphate [27] together with calcite on the beads’ surface. This taphonomic process is clearly observed on Fig 8 where a white layer (calcium and phosphate layer) overlays a reddish one. Indeed, it can be seen in Fig 8D that calcite coming from the cave has precipitated on the top of every layer.

This means that the outermost layer of the coating is the result of taphonomic process occurred since burial in the sediment and this is not an intentional coating layer. FTIR microscopy of the surface of this white core material showed additional bands to the υ_1_ symmetric stretching mode of the aragonite (*c*. 1087 cm^-1^) at *c*. 881, 1432 and 712 cm^-1^ that can be assigned to the vibrational active modes of calcite [25] (Fig 7B). This fully supports the above stated precipitation of calcite on the surface of the beads from Cova del Gegant.

However, the band assignment of μ-FTIR spectra collected on the bead surface coating and white core (Fig 7) proved challenging because spectra were poorly defined and too noisy. Although the appearance of the reddish yellow coating under microscope magnification resembles that of an amber, μ-FTIR spectra of the coating surface do not show the characteristic Baltic shoulder (succinite) nor the characteristic bands of Sicilian amber (simetite), Cretaceous Iberian amber, or any fossil resins archaeologically recorded. Thus, the signal comparison of the recorded spectra to natural resin reference spectra failed to give a positive match. However, it was quite clear under microscope magnification that the reddish-yellow coating was made of some sort of surface oxidized resin.

Natural resins and their type can be identified by their infrared spectral features. The diagnostic IR absorption bands for natural resins, as described by Table 1 in [28], are bands at *c*. 1695, 1448–1467, 1382–1387, 1178–1184, 1078–1092, 1028–1038 and 887–897 cm^-1^. These may be used to distinguish tree resins from shellac. All of these bands although present are hardly appreciated in Fig 7.

#### Natural terpenous resins

Apart from shellac, which is of insect origin, natural resins or terpenoids [according to [29] the term terpenoid is now preferred as the generic name for natural resins] exuded naturally from certain trees as viscous liquids which subsequently harden by evaporation of volatiles or the partial oxidative polymerization of some components [30] (Mills and White 1977, p. 13). Diterpenoids comprise the bulk composition of resins from the families Coniferae (Pinaceae, Cupressaceae, and Araucariaceae) and Leguminosae. The most abundant source of terpenous resins are trees of the genus *Pinus*.

**Fig 9:**
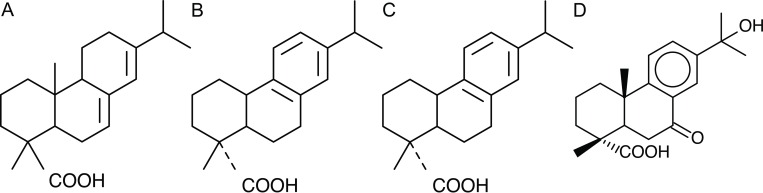
(A) Abietic acid structure. (B) Dehydroabietic acid structure. (C) 7-Oxodehydroabietic acid structure. (D) 15Hydroxy-7oxo-Dehydroabietic acid structure.

Diterpenoid resins possess mainly abietane, pimarane, and labdane skeletons. In non-polymerized structures abietane and pimarane compounds are predominant. The Pinaceae, and especially the genus *Pinus*, have resins with a high content of abietic acid (Fig 9), and a small number of abietane isomers [30], but resins from *Abies* and *Picea* species also contain large amounts of labdanes, as do Cupressaceae resins [29].

Terpenous resins are a complex mixture of molecules that changes considerably with time due to their high susceptibility to oxidation or polymerization. Their identification has the reputation of being a complex analytical task due to the broad range of oxidation products resulting from the oxidative ageing process observed [29–33]. In contrast, the first molecular changes to natural resins can be observed a few weeks after collection [32].

Terpenoids are susceptible to a number of alterations mediated by oxidation and reduction reactions as well as changes brought about by microbial activity [29]. For example, the ageing of diterpenoid resins at molecular level is predominantly the result of the oxidation / dehydrogenation of abietanes. Non-oxidized abietanes quickly convert to abietic acid (Fig 9A) and that to dehydroabietic acid (Fig 9B). Further oxidation leads to the formation of 7-oxodehydroabietic acid (Fig 9C) and 15-hydroxy-7-oxodehydroabietic acid (Fig 9D). Intermolecular reactions occurring between two carboxylic acid groups from abietanes or pimaranes give rise to the formation of acid anhydrides [34]. Although pimaranes are more stable, some studies also revealed their diminution during ageing [30, 32–36].

Besides, many studies of Pinaceae resin [this genus includes many species (*Pinus*, *Larix* and *Abies*), mainly trees, found mostly in the northern hemisphere. One of the features of *Pinus* species is that they exudate a terpenous resinous secretion [32]] made by FTIR Spectroscopy and Raman Spectroscopy indicate that resins of different species show spectra directly related to the precise amount of abietanes and a smaller portion of pimaranes [28, 32]. The dehydrogenation process in terpenous natural resins as well as the effects of soil pH after deposition must be considered when trying to identify the nature of the resin used for coating the beads. This requires quality spectra where the overlaps due to compound mixture other than those naturally occurring in resins are absent. Because of this, we decided to take a micro sample of the reddish-yellow coating to record spectra in FTIR transmission mode (KBr pellet) in the hope of acquiring spectra of the coating material not mixed with carbonates and phosphates recorded on bead surface.

#### Spectra discussion

**Fig 10:**
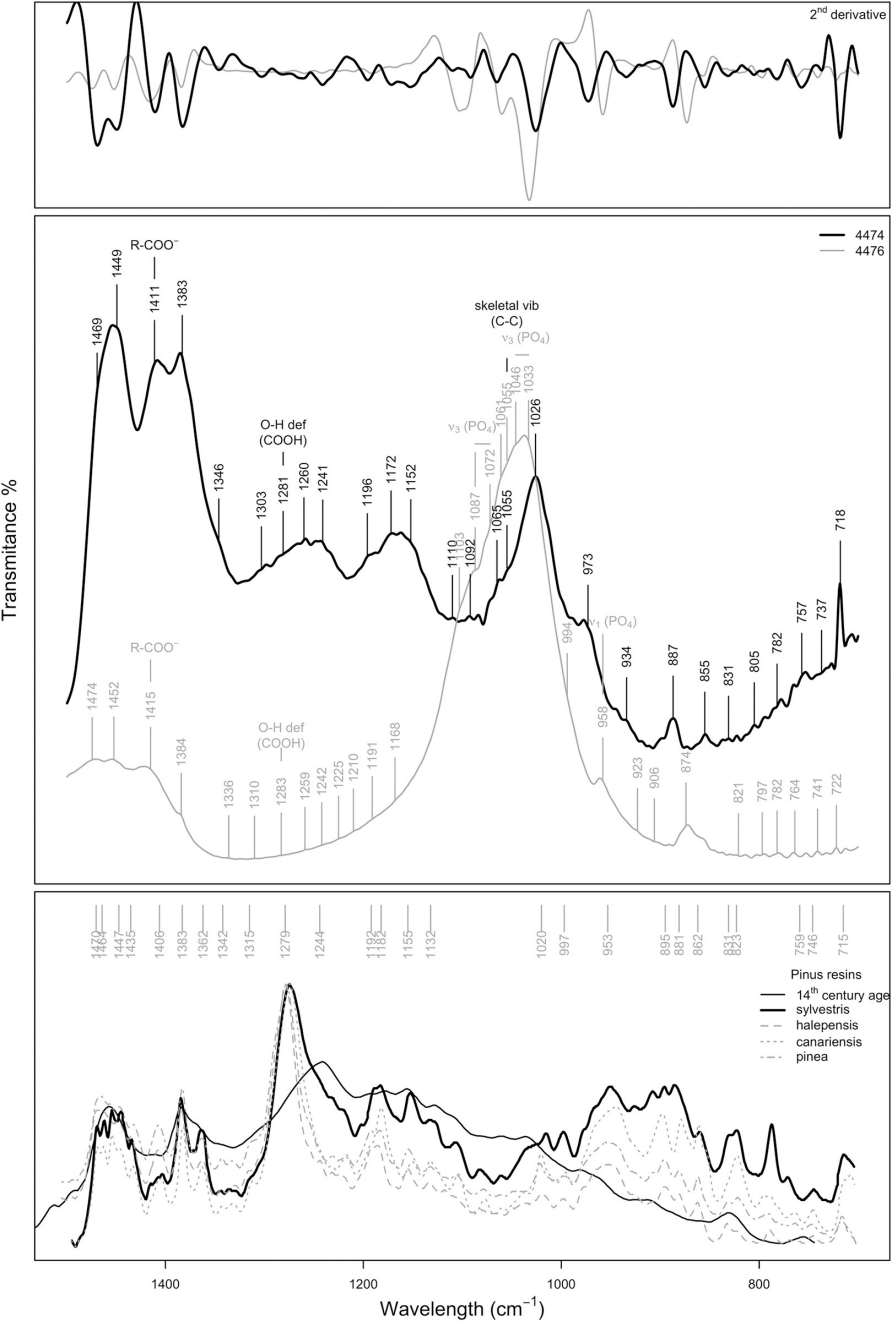
FTIR spectra in the diagnostic region (1500–700 cm^-1^). A) Second gap derivative of the spectra 4474 and 4476. B) 4474 and 4476 spectra, bands identified with the custom-built minimum/maxima identification function applied to the second gap derivative are labelled. C) Reference spectra discussed in the text are plotted, labels correspond to band position identified for 4474 and 4476 spectra.

The transmission spectra for specimen 4476 seem to be polluted by the post-depositional phosphate precipitation. Fortunately, that helped to clearly identify υ_1_ hydroxyapatite vibrational modes at *c*. 564 and 602 cm^-1^, in part due to the 200 cm^-1^ broader spectral range scanned by the DTGS detector of the FTIR transmission mode compared to the nitrogen-cooled MCT detector of the FTIR microscope. This supports the above-described post-depositional precipitation of apatitic calcium phosphate. In addition, the terpenous resin diagnostic region, between 1500 and 700 cm^-1^, is dominated by the presence of an intense and broad band where C-C skeletal vibrations of the abietanes (abietic/dehydroabietic acid) are overlapped by the υ_3_ PO_4_ vibrations of the post-depositional phosphates (Fig 10). However, the transmission mode recorded spectra showed better defined and less noisy bands, which clearly improved band assignment in the diagnostic region where most of the molecular bond vibrations occur. The presence of C-C skeletal vibrations of abietic/dehydroabietic acid, the most abundant molecules in aged resins [30, 35], points to the use of a natural terpenous resin to coat the bead. That is, the coating is made out of a tree resin that has undergone molecular transformation over time.

Fig 10 shows the FTIR spectra in the diagnostic region (1500–700 cm^-1^). In this figure the second derivative spectrum is plotted against the recorded spectra and reference spectra from [32] of different *Pinus* species resins and a 14^th^ century *Pinus* resin used as a painting varnish. The second derivative spectrum identified bands and molecular structure diagnostic bands are indicated over the samples’ recorded spectra. Literature identified bands for *Pinus halepensis* are indicated on the y upper axis. Band assignment to specific molecular structures was based primarily on the available literature about aged resins [32–33, 36, 39]. It is possible to observe a fairly good match between the *Pinus halepensis* fingerprint, the 14^th^ century *Pinus* resin and the recorded spectra, especially that of Sample 4474. However, some differences between the references and the recorded spectra can be appreciated (Fig 10).

According to [28, 32, 34] it is possible to first identify the resin class (e.g., tree, insect, wax) by their characteristic functional group vibration and second to identify the type of resin (Pinacae, Cupressaceae, Araucariaceae, Anacardiaceae…) by the amount of abietanes and of pimaranes in the spectra.

Here, the assignment of bands to specific molecular structures for resin type identification was based primarily on available literature on fresh and aged natural terpenous resins (see Table 2). However, although the match of at least half of the bands with the reference material is a strong indication of the type of resin, visual comparison of the sample spectrum with that of the reference material is mandatory to determine resin type [28].

**Table 2:** FTIR band assignments for Cova del Gegant samples.

Band (cm-1)	Band assignments	Reference
3400	O–H stretching, increases with aging	[32]
2936	C-H stretching	[28]
2929	C-H stretching *Pinus pinea*, *Pinus halepensis* ν(CH_2_), ν(CH_3_) in abietic acid	
2869	C-H stretching *Pinus pinea*, *Pinus halepensis* ν(CH_2_), ν(CH_3_) in abietic acid	[28,32,38]
2652	C-H stretching oxidized	[32]
2534	C-H stretching oxidized	[28, 32]
1823	ν(C = O) of abietic acid. This band is assigned to unsaturated ketones, which form as primary oxidation products by the decay of hydrogen peroxides.	[33]
1800sh	Acid anhydride groups formed by the reaction of two carboxylic acid groups from different molecules (abietanes or pimaranes)	[32]
1739	Acid anhydride groups formed by the reaction of two carboxylic acid groups from different molecules (abietanes or pimaranes)	[32]
1725sh	C = O stretching of the ketone group of the 7-oxodehydroabietic and 15-hydroxy-7oxo-dehydroabietic acids	[32]
1713	*Pinus halepensis* ν(C = O) of abietic acid	[39]
1695sh	C = O stretching (COOH) dehydroabietic acid	[28]
1690	C = O stretching group isopimaric acid	[34]
1685	ν(C = O) of abietic acid. This band is assigned to unsaturated ketones, which form as primary oxidation products by the decay of hydrogen peroxides.	[33]
1664	*Pinus halepensis* ν(C = O) of abietic acid	[32, 38]
1645	C = C stretching of isopimaric acid (Beltran et al. 2017), *Pinus halepensis* abietic acid ν(C = C) trans conjugated (Brody et al. 2002)	[32, 38]
1612	*Pinus halepensis* abietic acid ν(C = C) aromatic	[32, 38]
1607w	C = C stretching	[32]
1575	C = C stretching (ring) 15-hydroxy-7-oxodehydroabietic acid	[34]
1514	C = C stretching	[32]
1500	Abietic acid ν(C = C) aromatic	[32]
1469	C-H deformation bending dehydroabietic acid [34], *Pinus halepensis* abietic acid δ(CH_2_), δ(CH_3_) [38]	[34, 38]
1449	*Pinus halepensis* C-H deformation bending (ring) COOH	[34]
1415–1411	*Pinus halepensis* abietic acid δ(CH_2_), δ(CH_3_)	
1383	C-H bending	[28]
1337	*Pinus halepensis* abietic acid δ(CH_2_), δ(CH_3_)	[38]
1303	*Pinus halepensis* abietic acid δ(CH_2_), δ(CH_3_) twisting	[38]
1281	*Pinus halepensis* abietic acid δ(CH_2_), δ(CH_3_) twisting [38], Coupled C-O/O-H deformation [34]	[34, 38]
1260	C-C-O stretching (-OH)	[34]
1241	C-C-O stretching (-OH) O–H deformation in–COOH associated with 15-hydroxy-7-oxo-dehydroabietic acid (increase intensity with aging)	[34]
1191–1196	15-hydroxy-7-oxodehydroabietic acid skeletal vibrations	[34]
1187–1192	15-hydroxy-7-oxodehydroabietic acid skeletal vibrations [34], *Pinus halepensis* abietic acid ν(CC) ring breathing [38]	[32,34, 38]
1152	Acid anhydride groups formed by the reaction of two carboxylic acid groups from different molecules (abietanes or pimaranes)	[32, 34]
1138	*Pinus halepensis* abietic acid ν(CC) ring breathing	[38]
1130	*Pinus halepensis* abietic acid ν(CC) ring breathing	[28]
1020	Related to common compounds	[32, 34]
1107	*Pinus halepensis* abietic acid ν(CC) ring breathing	[38]
1028	C-H deformation (aromatic ring)	[34]
980	*Pinus halepensis* abietic acid ρ(CH_2_), ρ(CH_3_)	[38]
910	*Pinus halepensis* abietic acid	[28, 38]
887	Skeletal C-C stretching dehydroabietic (isopropil group) [34], *Pinus halepensis* abietic acid [38]	[32,34, 38]
823		[28]
718	Skeletal vibration abietic/dehydroabietic [32, 34], ν(CC) isolated [38]	[32, 34, 38]

In the untransformed recorded transmittance spectra, band positions are difficult to identify due to occurring overlaps. Therefore, spectra second gap derivatives were used for band recognition using the gap derivative tool of R‘s prospect package [37] and a custom built minimum/maxima identification function.

The main compounds observed for Samples 4474 and 4476, in addition to PO_4_ for specimen 4476, are oxidized abietanes. Whereas the main spectral features identified below 1500 cm^-1^ are due to C-H, C-O and C-C wags and bends of the abietanes aging products, for example, hydroabietic acid and 15-hydroxy-7-oxo-dehydroabietic acid. However, other compounds in minor amounts are also present in the recorded spectra. Bands at *c*. 1182 and 1020 cm^−1^ are probably related to common compounds, and the 1020 cm^−1^ band is proposed as a pimarane marker (CH_2_ = CH wagging) [32].

The strongest absorptions in specimen 4474 between 1065 and 963 cm^-1^ are related to the skeletal vibrations of oxidized abietanes: abietic, dehydroabietic acid, 15-hydroxy-7-oxodehydroabietic acid… However, strong absorbance bands assigned to C-H bending modes occur at *c*. 1469, 1649, 1383 and 1028 cm^-1^ [28, 32, 34], and moderate to weak absorbance at 1281 and 1245 cm^-1^, are assigned to the coupled C-O/O-H deformation associated with the common oxidized abietane structure [34]. The weak bands at *c*. 1191–1196, 887 and 718 cm^-1^ are assigned to 15-hydroxy-7- oxodehydroabietic acid skeletal vibrations. Furthermore, according to [32, 34], the band at 1260, 1241 and 1152 cm^-1^ is specific to acid anhydride groups formed with aging by the reaction of two carboxylic acid groups from different molecules (abietanes or pimaranes).

**Fig 11:**
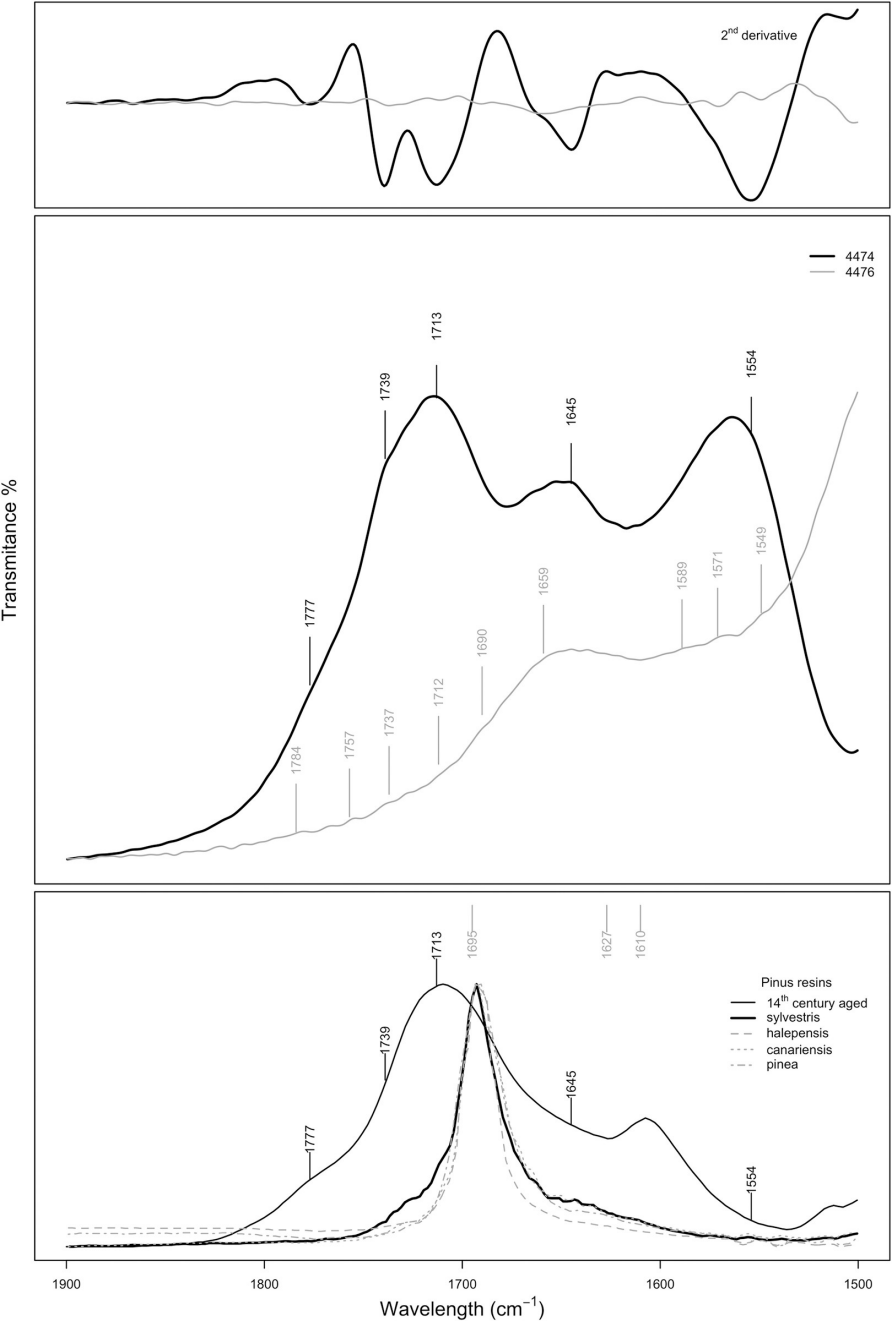
FTIR spectra in the carbonyl region.

The C = C and C = O region (1900–1500 cm^-1^) is where terpenous resins show the strongest hydrocarbon and carbonyl-stretching band (Fig 11). In this region multiple overlaps from the various resin aging products occurs [33]. For example, a broad band formed by the overlap of the ν(C = O) associated with the ketone group of the 7-oxodehydroabietic and 15-hydroxy-7oxo-dehydroabietic acids at *c*. 1725 cm^-1^ [32], the ν(C = O) at *c*. 1713 cm^-1^ [39], the ν(C = O) in dehydroabietic acid at *c*. 1695 cm^-1^ [28] and the ν(C = O) of abietic acid at *c*. 1685 cm^-1^ [33]. The latter band is assigned, according to [33], to unsaturated ketones, which form as primary oxidation products by the decay of hydrogen peroxides. Additionally, the presence of bands peaking at *c*. 1690 and 1645 cm^-1^ associated with the ν(C = O) and ν(C = H) in isopimaric acid [34], the bands at *c*. 1575 cm^-1^ assigned to the C = C stretching in 15-hydroxy-7- oxodehydroabietic acid, the band centred at *c*. 1823 cm^-1^ related to ν(C = O) of abietic acid, and the bands at *c*. 1612 and 1500 cm^−1^ related to the aromatic groups, agrees with the identification of those acids.

**Fig 12:**
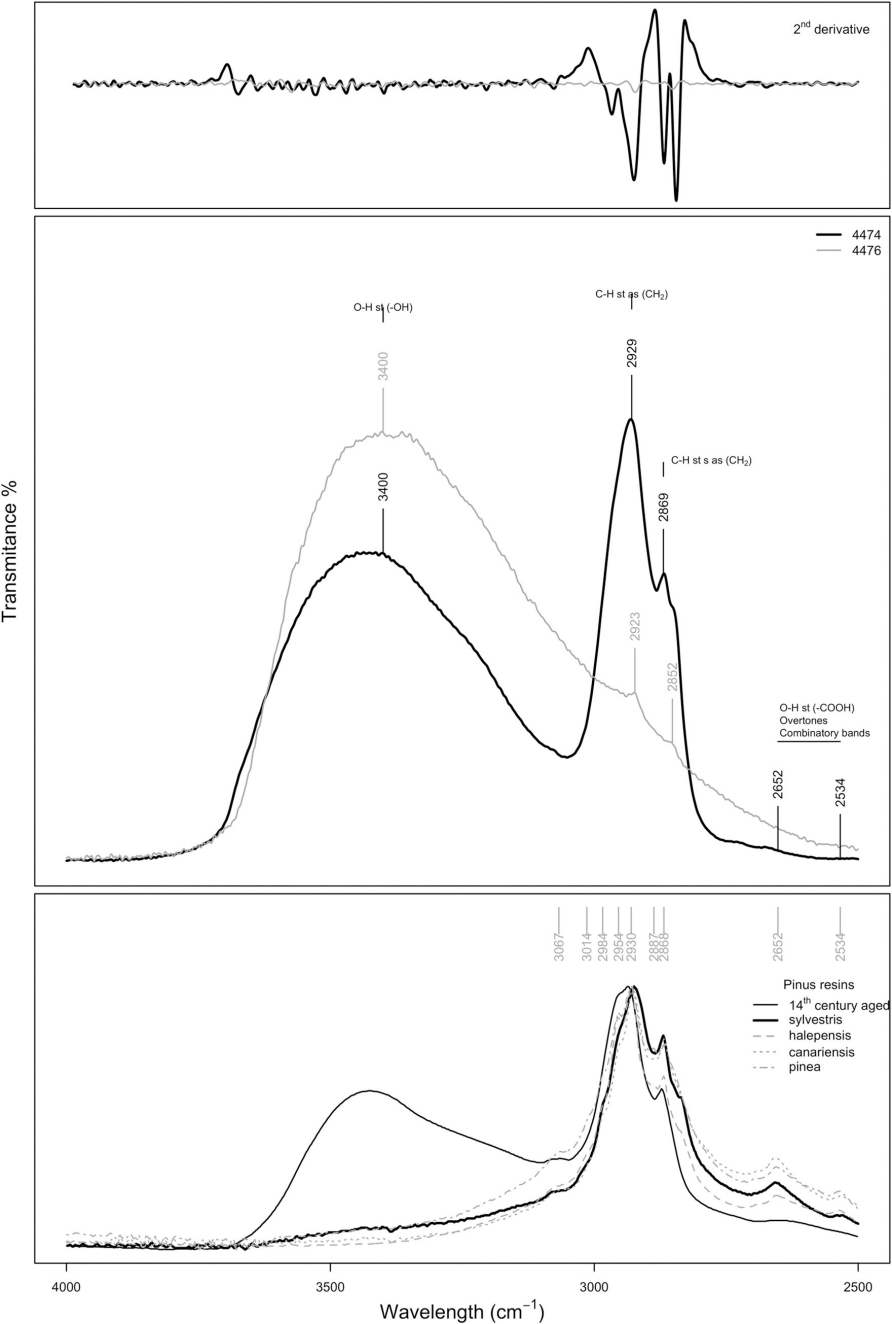
FTIR spectra in the OH region.

Finally, in Fig 12 is shown the C-H and O-H stretching region (4000–2500 cm^-1^), where it is possible to observe the bands associated with the hydroxyl groups at *c*. 3400, 2652 and 2530 cm^−1^ (O–H stretching) and the C-H stretching at c. 2936, 2929, 2869, 2652 and 2534 cm^−1^ of aged resins.

The identification of the main characteristic bands of abietane and primarane aging products, for example, abietic, dehydroabietic, 7-oxodehydroabietic, 15-hydroxy-7oxo-dehydroabietic, and isopimaric acid, points to the use of a tree resin, most probably of the *Pinus* genus. Additionally, the partial match of the spectral features of the recorded samples in the diagnostic region with those of 14^th^ century aged *Pinus* resin reference material and the identification of *Pinus halepensis* in Level XXV charcoals (anthracological analysis by E. Allué -personal communication) might point to the use of an exudate of *Pinus halepensis*.

### La Molina (C17)

**Fig 13:**
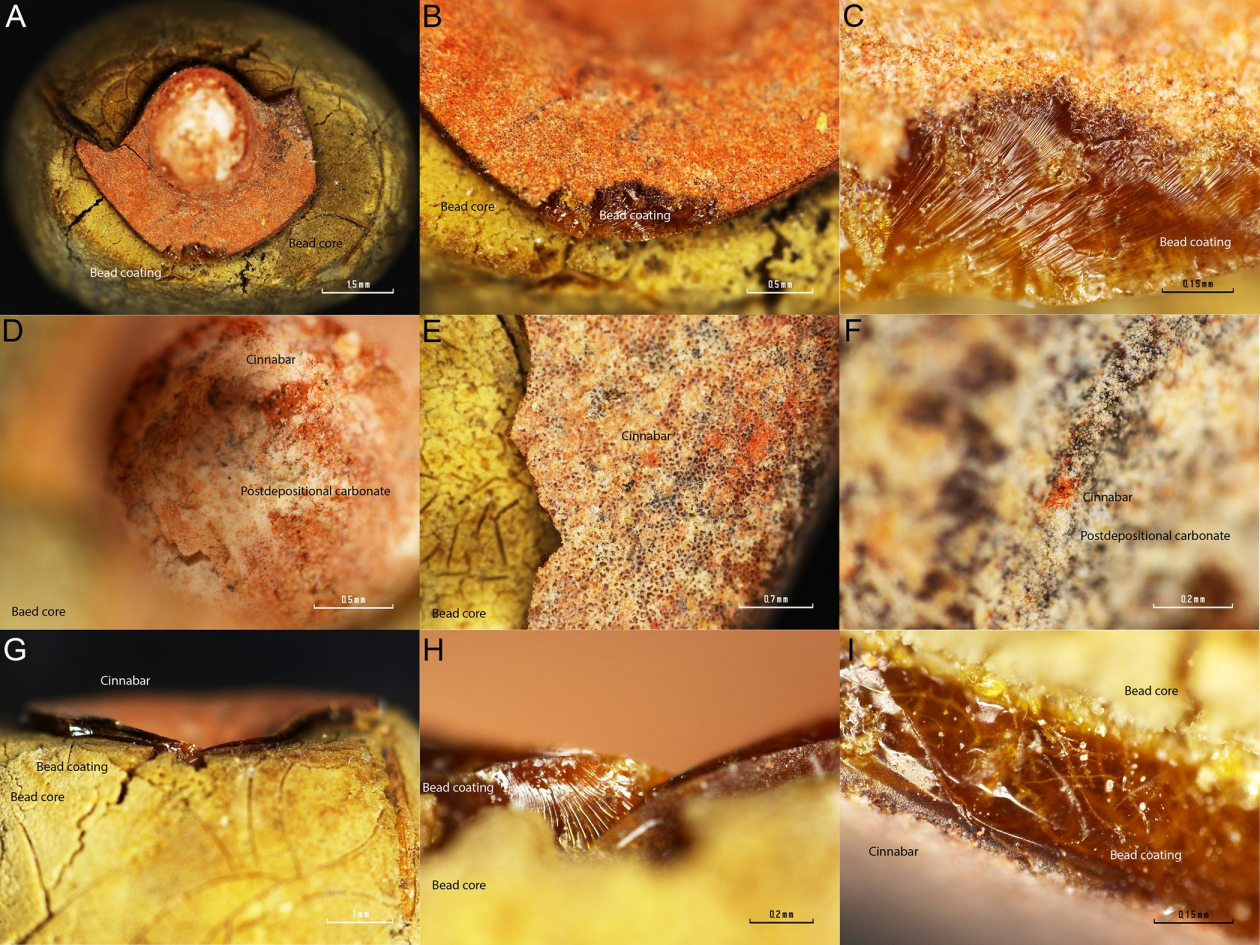
Detailed microphotographs of La Molina’s beads.

Observation under microscope shows that a thin white and red layer is on the top of the bead coating (Fig 13). Unlike the Cova del Gegant reddish-yellow beads, the La Molina beads’ pinkish-red in appearance is most likely due to a post-depositional cinnabar (HgS) layer. It seems that the red pigment is somehow dispersed over a white matrix that is all over the bead surface, and that this white material is clogging the bead perforation. Therefore, the bead coating stratigraphy consists of the bead core, an amber-like resin, a white matrix and a red pigmentation layer. The white layer is interpreted as a post-depositional layer formed over time. On top of this post-depositional white layer, a red pigment sprinkled all over the bead surface can be observed. Although the beads were not found within a stratigraphy, this fact seems to indicate that the beads belong to the first inhumation phase and therefore they are not associated with the female individual (E1), the last individual to be buried in the tomb [18]. Opening a possible association of the beads with one of the two male individuals buried in the earlier time of use of this artificial funerary cave.

Chemical analysis performed with an EDX handheld device on the bead surface shows that it is mainly composed by calcium, *c*. 70 atomic %, and in a minor amount of sulphur and mercury (Table 3), i.e., cinnabar (mercury sulphide). Although it has been recorded at other sites that cinnabar can be diluted with iron oxides [40] or calcium phosphates [41], this seems not to be the case. However, XRD and DcμRS have been performed on the sample surface to precisely characterize the red pigment.

**Table 3:** EDX analysis on the La Molina beads’ coatings expressed as atomic percentage.

	Al	Si	P	S	Cl	Ca	Ti	Mn	Fe	Rb	Sr	Mo	Sn	Sb	Au	Hg
C17-1007-90	8.4	16.3	0.6	4.6	2.3	66.4	0.05	0.52	0.46	0.01	0.025	0.00	0.01	0.01	0.16	0.16
C17-1007-89	7.6	2.2	0.6	4.2	5.2	79.4	0.00	0.17	0.22	0.02	0.06	0.01	0.00	0.00	0.22	0.22

**Fig 14:**
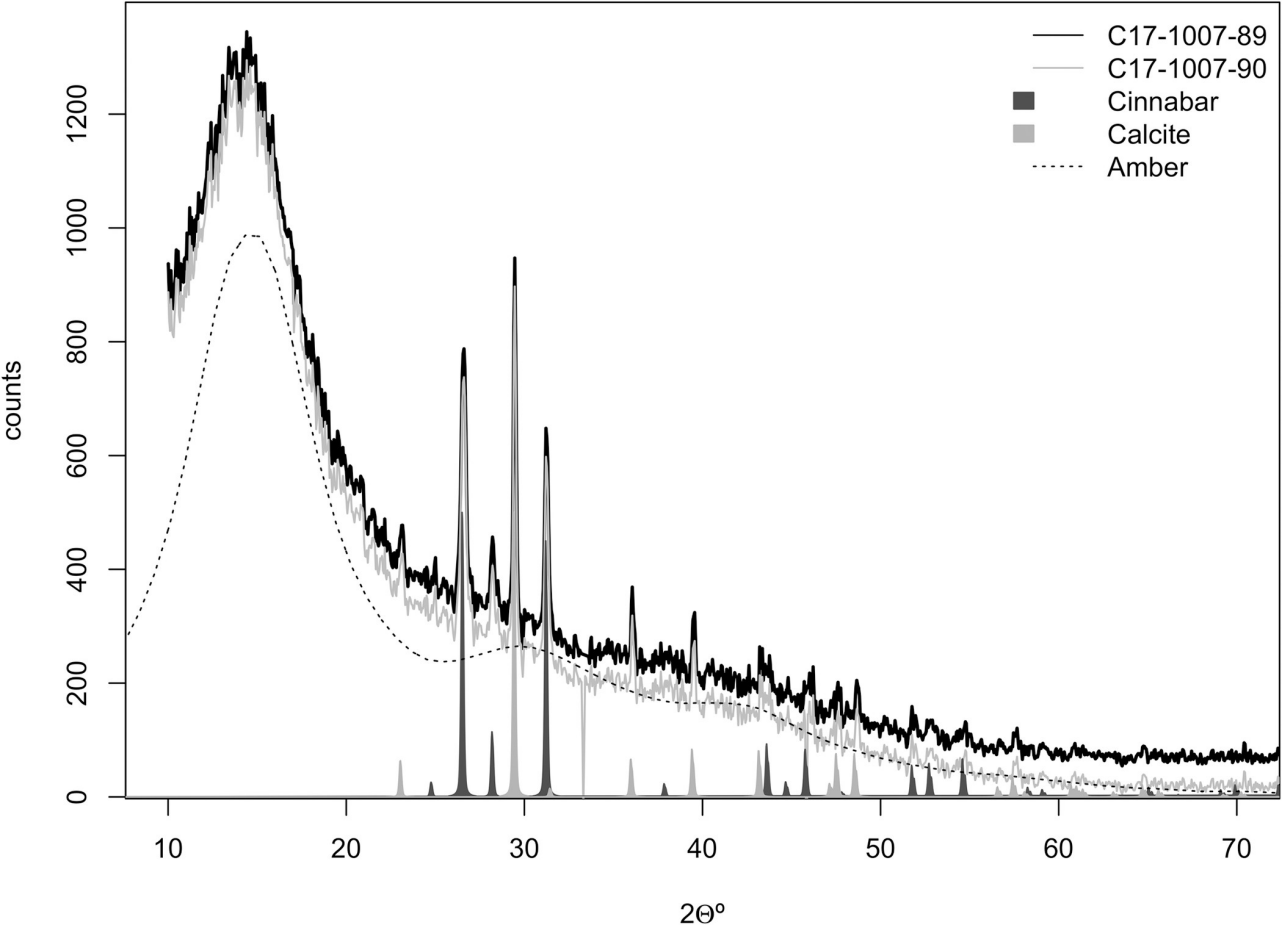
X ray powder diffraction pattern of La Molina samples coatings compared to cinnabar, calcite and amber patterns.

**Fig 15:**
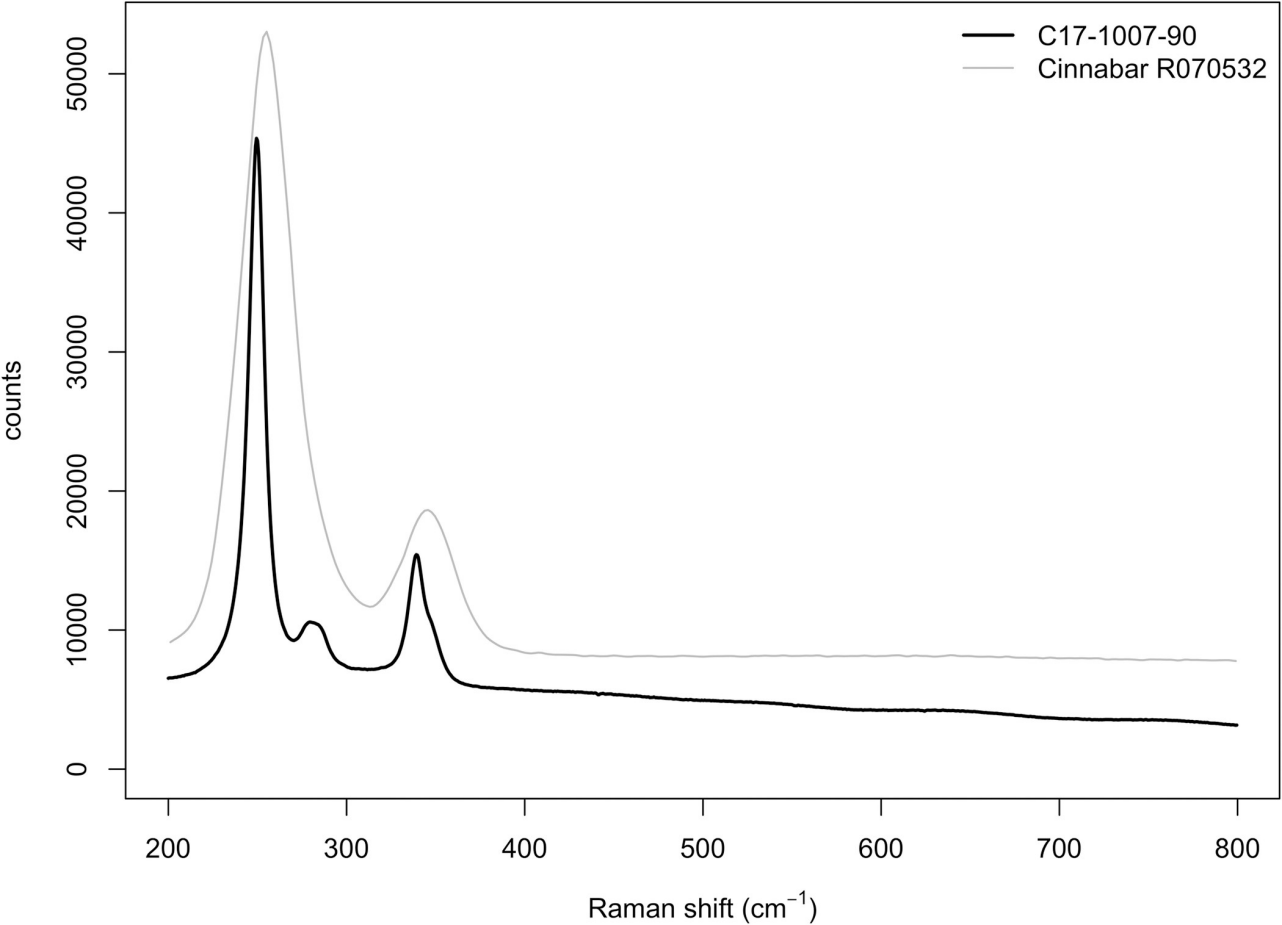
Raman spectra of La Molina samples coatings compared to the cinnabar reference spectrum (www.rruff.info, R070532).

XRD analysis (Fig 14) shows that the bead red coating is formed by a mixture of calcite, cinnabar and an oxidized and/or polymerized abietane. The broad band peaking between 14.3 to 15.55° 2θ in the diffractogram has been associated with oxidized and/or polymerized abietanes [42]. Therefore, XRD analysis shows that the beads’ coating is formed by calcite, cinnabar and an oxidized abietane, most likely a natural terpenous resin. Indeed, DcμRS yielded the typical cinnabar spectrum (Fig 15).

Unlike Cova del Gegant, La Molina samples do not display bands that can be associated with isopimaric acid or with the formation of anhydride groups by the reaction of carboxylic acids groups. This is most likely due to the different pH conditions of the burial sediment. Nevertheless, the FTIR spectra in the carbonyl and diagnostic regions (Table 4) show bands compatible with those of abietane aging products, for example abietic, dehydroabietic, 7-oxodehydroabietic and 15-hydroxy-7-oxo-dehydroabietic acids, that is, to a tree resin.

**Table 4:** Band assignments of La Molina´s beads coating.

Band (cm-1)	Band assignments	Reference
1725sh	C = O stretching of the ketone group of the 7-oxodehydroabietic and 15-hydroxy-7oxo-dehydroabietic acids	[32]
1709	*Pinus halepensis* ν(C = O) of abietic acid	[39]
1664	*Pinus halepensis* ν(C = O) of abietic acid	[38]
1649	C = C stretching of isopimaric acid [34], *Pinus halepensis* abietic acid ν(C = C) trans conjugated [38]	[34, 38]
1610	*Pinus halepensis* abietic acid ν(C = C) aromatic	[32, 38]
1607w	C = C stretching	[32]
1563	*Pinus halepensis* abietic acid ν(C = C)	[34]
1469	C-H deformation bending dehydroabietic acid [34], *Pinus halepensis* abietic acid δ(CH_2_), δ(CH_3_) [38]	[34, 38]
1443	*Pinus halepensis* C-H deformation bending (ring) COOH	[34]
1415	*Pinus halepensis* abietic acid δ(CH_2_), δ(CH_3_)	
1383	C-H bending	[28]
1332	*Pinus halepensis* abietic acid δ(CH_2_), δ(CH_3_)	[38]
1303	*Pinus halepensis* abietic acid δ(CH_2_), δ(CH_3_) twisting	[38]
1281	*Pinus halepensis* abietic acid δ(CH_2_), δ(CH_3_) twisting [38], Coupled C-O/O-H deformation [34]	[34, 38]
1188	15-hydroxy-7-oxodehydroabietic acid skeletal vibrations (Beltran et al. 2017), *Pinus halepensis* abietic acid ν(CC) ring breathing [38]	[23, 34, 38]
1182	*Pinus halepensis* abietic acid ν(C = C) ring breathing	[38]
1152	Acid anhydride groups formed by the reaction of two carboxylic acid groups from different molecules (abietanes or pimaranes)	[32, 34]
1139	*Pinus halepensis* abietic acid ν(CC) ring breathing	[38]
1107	*Pinus halepensis* abietic acid ν(CC) ring breathing	[38]
1028	C-H deformation (aromatic ring)	[34]
981	*Pinus halepensis* abietic acid ρ(CH_2_), ρ(CH_3_)	[38]
887	Skeletal C-C stretching dehydroabietic (isopropil group) (Beltran et al. 2017), *Pinus halepensis* abietic acid	[32, 34, 38]
718	Skeletal vibration abietic/dehydroabietic (Beltran et al. 2016, 2017), ν(CC) isolated [38]	[32, 34, 38]

## Discussion

The use of beads covered by tree resin has been documented for the first time at the artificial cave of La Molina (Lora de Estepa, Sevilla) and Cova del Gegant (Sitges, Barcelona), dated in the third and second millennia BC respectively.

Organic materials easily acquired and available in the environment were used both for the cores and the coating. This is especially clear in the way *Cardium* shells were used at the coastal site of Cova del Gegant and most likely seeds at La Molina. Similarly, pines are abundant in the whole Mediterranean basin and in the Iberian Peninsula in general.

In our opinion, the ultimate goal of using this technology was to imitate the organoleptic properties of amber. With this type of surface coating it was possible to emulate effectively the translucence, shine and colour that made amber such an appreciated material. The use of translucent reddish-yellow minerals, aesthetically similar to amber and possible substitutes for amber, has been documented from the late fourth millennium BC [2]. Many reasons may have led to this use of such amber equivalents as citrine quartz and carnelian. One of them may lie in the insufficient supply of amber to meet an increasing demand. The dynamic of change towards increasingly complex and unequal societies accelerated greatly in the early third millennium BC and as a result the number of exotic items accumulated around important individuals with a different or special treatment in both life and death [6, 43]. However, few raw materials look like amber and they are equally unusual in the archaeological record. Therefore, in a situation of increasing demand it is logical to imagine that alternatives were found in the coatings, particularly in cases of scarcity of those raw materials.

The increase in demand and the need for exotic elements to be exhibited by certain individuals is clear in burials like those at Anta Grande do Zambujeiro (Évora, Portugal) and the *tholos* at Montelirio (Castilleja de Guzmán, Seville) which concentrate nearly 90% (in weight and number of items) of amber in the Iberian Peninsula in the third millennium BC [2].

In this respect, the large amount of ivory and cinnabar in the hypogean tomb of La Molina seems to reinforce the idea that the individuals in the most exclusive sector of the collective tomb were able to acquire exotic raw materials. It is however strange that individuals who possessed ivory and cinnabar were unable to obtain amber, at a time when the Sicilian sources had not yet become exhausted and Sicilian amber was reaching the southern Iberian Peninsula. Perhaps the individuals at La Molina were less able to acquire amber than those buried in the *tholos* of Montelirio (Castilleja de Guzmán, Sevilla) due to the high demand or cost, or simply the exchange networks of which they formed part did not have access to amber. However, ivory and Sicilian amber may have been arriving together through North Africa, so the scarcity of amber compared with ivory and its association with wealthy burials may indicate that amber enjoyed greater social value as it was a scarce raw material (always based on the proportion of the number of items in each category).

In this context, the amber equivalents may have been: i) a substitute to meet the high demand for that raw material which was impossible to satisfy by the suppliers; ii) a low-cost product with the same social function as amber used by segments of society that were not wealthy enough to acquire the real product; iii) products used by middlemen to cheat the purchasers. The last possibility might be the case in Cova del Gegant where the four resin-covered beads were found together with two beads of Sicilian amber [2] of very similar size and shape. This supports the idea that the resin-covered beads can be regarded as amber equivalents as there are no apparent differences between them and they form a homogeneous and coherent group of adornments *de visu*.

The identification of those two beads as Sicilian amber [2] poses a problem regarding the interaction networks of the Bronze Age groups. Unlike the situation in the third millennium BC when most of the amber in the Iberian Peninsula came from Sicily, most of the second millennium BC beads came from the Baltic region [2]. At that time, the sources of Sicilian amber were exhausted, and the central and eastern Mediterranean were the poles of attraction for most of the exotic products that were circulating in Europe and the Mediterranean. Thus, the epicentre of exchange networks moved towards the central and eastern Mediterranean while the source of the materials changed. This would have led to a lack of supply in the Iberian Peninsula, which would be reached by very little amber, mostly from the Baltic. It is therefore significant that Sicilian amber reached Cova del Gegant, possibly some of the last Sicilian batches, together with other elements clearly of a southern origin, such as the gold *tutuli*, probably through exchange networks that had been operative in the previous millennium. Again, the presence of the two gold *tutuli* in the mortuary level shows that the inhumed individuals had been able to acquire exotic elements. Therefore, as no doubts can be harboured about the wealth of the individuals, it is possible that the middlemen who supplied the elite with their prestige items, in a situation of shortages in supplies, high demand, exhaustion of traditional sources and the inability to compete with the central and eastern Mediterranean decided to create the amber-equivalent beads. However, it is also possible that, owing to the difficult access to this prestigious raw material, the two Sicilian amber beads were re-used and come from earlier burials, and the resin-covered beads were produced locally to increase the number of items for display.

In summary, the use of resin-covered beads, that has never been documented before, was an alternative developed in areas with a high capacity of acquiring exotic raw materials. However, in the case of La Molina the buried individuals did not possess amber or any other elements of personal adornment, and at Cova del Gegant the buried individuals formed part of eccentric dynamics in the acquisition of amber elements. These amber-equivalent beads attest the technical and symbolic complexity in the choice of raw materials in Late Prehistory and the search for alternatives in times when it was impossible to acquire a particular raw material.

These two archaeological sites resemble each other in many ways despite the geographic and chronological differences and point to technical practices and knowledge in Late Prehistory that would have been more frequent than tends to be documented, as other finds in the Near East have shown. In the Levant, this practice has been well documented in the case of the manufacture of artificial turquoise beads [14, 16]. It is quite likely that many of these adornments with artificial coatings have not been preserved.

The identification of amber beads at Iberian mortuary sites *de visu*, without FTIR analysis to verify if they are made of fossil amber or of resin, may lead to erroneous identifications and over-represent the presence of amber.
